# Supplementary material for: Multi‐dataset identification of innovative feature genes and molecular mechanisms in keratoconus
Source: J Cell Mol Med. 2024 Sep 19;28(18):e70079. doi: 10.1111/jcmm.70079 (PMC11412914; doi:10.1111/jcmm.70079)
Supplement: Supplementary file 2 — Material S2. [file JCMM-28-e70079-s002.zip › 7.GSEA analysis/2.ATOH7/KEGG_RIBOSOME.html]

Details for gene set KEGG\_RIBOSOME[GSEA]

|  || Dataset | ATOH7.group.cls#h\_versus\_l |
| Phenotype | group.cls#h\_versus\_l |
| Upregulated in class | l |
| GeneSet | KEGG\_RIBOSOME |
| Enrichment Score (ES) | -0.2508371 |
| Normalized Enrichment Score (NES) | -0.9318489 |
| Nominal p-value | 0.5042373 |
| FDR q-value | 1.0 |
| FWER p-Value | 1.0 |
Table: GSEA Results Summary

  

Fig 1: Enrichment plot: KEGG\_RIBOSOME      
 Profile of the Running ES Score & Positions of GeneSet Members on the Rank Ordered List

  

| SYMBOL | TITLE | RANK IN GENE LIST | RANK METRIC SCORE | RUNNING ES | CORE ENRICHMENT || 1 | RPL28 | na | 8690 | 0.105 | -0.2083 | Yes |
| 2 | RPS21 | na | 9804 | 0.084 | -0.2061 | Yes |
| 3 | RPS24 | na | 10728 | 0.070 | -0.2041 | Yes |
| 4 | RSL24D1 | na | 11081 | 0.065 | -0.1879 | Yes |
| 5 | RPL3 | na | 12412 | 0.047 | -0.2070 | Yes |
| 6 | RPS27L | na | 12671 | 0.044 | -0.1966 | Yes |
| 7 | RPS26 | na | 12706 | 0.044 | -0.1799 | Yes |
| 8 | RPL37 | na | 13066 | 0.040 | -0.1741 | Yes |
| 9 | RPL9 | na | 13274 | 0.038 | -0.1647 | Yes |
| 10 | RPL15 | na | 13429 | 0.036 | -0.1546 | Yes |
| 11 | RPS27A | na | 13626 | 0.034 | -0.1463 | Yes |
| 12 | RPS5 | na | 13746 | 0.033 | -0.1363 | Yes |
| 13 | UBA52 | na | 13825 | 0.032 | -0.1255 | Yes |
| 14 | RPL18A | na | 13853 | 0.032 | -0.1132 | Yes |
| 15 | RPL13A | na | 13886 | 0.032 | -0.1013 | Yes |
| 16 | RPS3A | na | 13946 | 0.031 | -0.0904 | Yes |
| 17 | RPS3 | na | 13955 | 0.031 | -0.0780 | Yes |
| 18 | RPS19 | na | 14045 | 0.030 | -0.0684 | Yes |
| 19 | RPL29 | na | 14121 | 0.029 | -0.0586 | Yes |
| 20 | RPL10A | na | 14160 | 0.029 | -0.0479 | Yes |
| 21 | RPL35A | na | 14168 | 0.029 | -0.0364 | Yes |
| 22 | RPS2 | na | 14204 | 0.029 | -0.0258 | Yes |
| 23 | RPL5 | na | 14389 | 0.027 | -0.0202 | Yes |
| 24 | RPL23A | na | 14422 | 0.026 | -0.0103 | Yes |
| 25 | RPS11 | na | 14459 | 0.026 | -0.0008 | Yes |
| 26 | RPS16 | na | 14462 | 0.026 | 0.0098 | Yes |
| 27 | RPL18 | na | 14470 | 0.026 | 0.0201 | Yes |
| 28 | RPL21 | na | 14515 | 0.026 | 0.0293 | Yes |
| 29 | RPS29 | na | 14688 | 0.024 | 0.0340 | Yes |
| 30 | RPL7 | na | 14701 | 0.024 | 0.0433 | Yes |
| 31 | RPL13 | na | 14762 | 0.023 | 0.0509 | Yes |
| 32 | RPL7A | na | 14764 | 0.023 | 0.0603 | Yes |
| 33 | RPL10L | na | 14829 | 0.023 | 0.0676 | Yes |
| 34 | RPL30 | na | 14964 | 0.022 | 0.0725 | Yes |
| 35 | RPL26L1 | na | 14995 | 0.021 | 0.0803 | Yes |
| 36 | RPL19 | na | 14998 | 0.021 | 0.0889 | Yes |
| 37 | RPS10 | na | 15033 | 0.021 | 0.0964 | Yes |
| 38 | RPLP0 | na | 15082 | 0.020 | 0.1033 | Yes |
| 39 | RPL17 | na | 15111 | 0.020 | 0.1107 | Yes |
| 40 | RPS15 | na | 15114 | 0.020 | 0.1188 | Yes |
| 41 | RPS17 | na | 15148 | 0.020 | 0.1260 | Yes |
| 42 | RPL35 | na | 15194 | 0.019 | 0.1326 | Yes |
| 43 | RPL41 | na | 15200 | 0.019 | 0.1403 | Yes |
| 44 | RPLP1 | na | 15207 | 0.019 | 0.1481 | Yes |
| 45 | RPL24 | na | 15286 | 0.019 | 0.1534 | Yes |
| 46 | RPS25 | na | 15319 | 0.018 | 0.1599 | Yes |
| 47 | RPL37A | na | 15378 | 0.018 | 0.1654 | Yes |
| 48 | RPL22L1 | na | 15558 | 0.016 | 0.1669 | Yes |
| 49 | RPS23 | na | 15618 | 0.016 | 0.1717 | Yes |
| 50 | RPS13 | na | 15683 | 0.015 | 0.1760 | Yes |
| 51 | RPL22 | na | 15685 | 0.015 | 0.1822 | Yes |
| 52 | RPL34 | na | 15695 | 0.015 | 0.1881 | Yes |
| 53 | MRPL13 | na | 15778 | 0.015 | 0.1916 | Yes |
| 54 | RPL4 | na | 15842 | 0.014 | 0.1956 | Yes |
| 55 | RPS15A | na | 15882 | 0.014 | 0.2001 | Yes |
| 56 | RPS8 | na | 15966 | 0.013 | 0.2030 | Yes |
| 57 | RPS18 | na | 16096 | 0.012 | 0.2043 | Yes |
| 58 | RPL31 | na | 16128 | 0.012 | 0.2082 | Yes |
| 59 | RPL38 | na | 16473 | 0.009 | 0.2019 | Yes |
| 60 | RPS12 | na | 16512 | 0.009 | 0.2043 | Yes |
| 61 | RPS28 | na | 16619 | 0.008 | 0.2044 | Yes |
| 62 | RPL27 | na | 16639 | 0.008 | 0.2070 | Yes |
| 63 | RPL6 | na | 16825 | 0.006 | 0.2041 | Yes |
| 64 | RPS7 | na | 16835 | 0.006 | 0.2063 | Yes |
| 65 | FAU | na | 16854 | 0.006 | 0.2082 | Yes |
| 66 | RPLP2 | na | 16973 | 0.005 | 0.2067 | Yes |
| 67 | RPL8 | na | 17150 | 0.003 | 0.2029 | Yes |
| 68 | RPL14 | na | 17235 | 0.002 | 0.2014 | Yes |
| 69 | RPL11 | na | 17262 | 0.002 | 0.2015 | Yes |
| 70 | RPL26 | na | 17736 | -0.002 | 0.1886 | Yes |
| 71 | RPL39 | na | 17858 | -0.003 | 0.1862 | Yes |
| 72 | RPS27 | na | 17975 | -0.004 | 0.1844 | Yes |
| 73 | RPL23 | na | 18004 | -0.004 | 0.1853 | Yes |
| 74 | RPS20 | na | 18015 | -0.004 | 0.1867 | Yes |
| 75 | RPL36 | na | 18079 | -0.005 | 0.1867 | Yes |
| 76 | RPL12 | na | 18142 | -0.005 | 0.1871 | Yes |
| 77 | RPL36A | na | 18708 | -0.010 | 0.1750 | Yes |
| 78 | RPS6 | na | 18999 | -0.013 | 0.1718 | Yes |
| 79 | RPS9 | na | 19747 | -0.019 | 0.1581 | Yes |
| 80 | RPL36AL | na | 20308 | -0.025 | 0.1521 | Yes |
| 81 | RPS4X | na | 23154 | -0.057 | 0.0931 | Yes |
| 82 | RPL32 | na | 23894 | -0.068 | 0.0995 | Yes |
| 83 | RPL3L | na | 26831 | -0.122 | 0.0642 | Yes |
| 84 | RPL27A | na | 26986 | -0.125 | 0.1106 | Yes |
| 85 | RPS4Y1 | na | 31682 | -0.277 | 0.0879 | Yes |
Table: GSEA details [plain text format]

  

Fig 2: KEGG\_RIBOSOME      
 Blue-Pink O' Gram in the Space of the Analyzed GeneSet

  

Fig 3: KEGG\_RIBOSOME: Random ES distribution      
 Gene set null distribution of ES for **KEGG\_RIBOSOME**
